# “Just” accuracy? Procedural fairness demands explainability in AI-based medical resource allocations

**DOI:** 10.1007/s00146-022-01614-9

**Published:** 2022-12-21

**Authors:** Jon Rueda, Janet Delgado Rodríguez, Iris Parra Jounou, Joaquín Hortal-Carmona, Txetxu Ausín, David Rodríguez-Arias

**Affiliations:** 1 Department of Philosophy 1, University of Granada, Granada, Spain; 2 University of Granada, Granada, Spain; 3 Universitat Autònoma de Barcelona, Barcelona, Spain; 4 Andalusian Health System, University of Granada, Granada, Spain; 5 Institute of Philosophy, Spanish National Research Council, Madrid, Spain; 6 FiloLab Scientific Unit of Excellence, University of Granada, Granada, Spain

**Keywords:** Artificial intelligence, Distributive justice, Explainability, Medical AI, Procedural fairness

## Abstract

The increasing application of artificial intelligence (AI) to healthcare raises both hope and ethical concerns. Some advanced machine learning methods provide accurate clinical predictions at the expense of a significant lack of explainability. Alex John London has defended that accuracy is a more important value than explainability in AI medicine. In this article, we locate the trade-off between accurate performance and explainable algorithms in the context of distributive justice. We acknowledge that accuracy is cardinal from outcome-oriented justice because it helps to maximize patients’ benefits and optimizes limited resources. However, we claim that the opaqueness of the algorithmic black box and its absence of explainability threatens core commitments of procedural fairness such as accountability, avoidance of bias, and transparency. To illustrate this, we discuss liver transplantation as a case of critical medical resources in which the lack of explainability in AI-based allocation algorithms is procedurally unfair. Finally, we provide a number of ethical recommendations for when considering the use of unexplainable algorithms in the distribution of health-related resources.

## Introduction

Traditionally, progress in clinical practice has been made through the systematic study of accumulated experience and statistical analysis of reduced amounts of data. Artificial intelligence (AI) provides new opportunities for the development of evidence-based medicine by simultaneously observing and rapidly processing an almost unlimited number of medical and nonmedical (sociodemographic, ethnic, geographical) inputs that could contribute to describe, explain and foresee health and disease (Buch et al. 2018; Ellahham et al. 2020). All of this can save time, energy, and money, especially in the diagnosis and management of patients. Machine Learning (ML) has also been applied to reinforce safety by minimizing risks and reducing the uncertainty about harmful events (Ellahham et al. 2020). The application of AI has achieved a revolutionary change in radiological diagnosis, for instance, by improving the accuracy of image analysis in the early detection of breast pathologies (Robertson et al. 2018). Patient triage is another area in which AI has been introduced, through wearable devices designed to monitor remotely and analyse vital signs—e.g., consciousness. In these AI systems, algorithms are trained to classify disease conditions based on severity, which helps predict survival in the pre-hospital environment (Kim et al. 2018; Ellahham et al. 2020), as well as in emergency departments through electronic triage (e-triage) (Levin et al. 2018; Ellahham et al. 2020), and in Intensive Care Units (ICUs) (Che et al. 2016; Nanayakkara et al. 2018).

Much of the excitement around medical AI is due to the high accuracy of these models. Advanced AI systems enable diagnoses, prognoses, and clinical recommendations based on numerous variables, which allows tailoring medical decisions to the specificities of each patient. Unfortunately, the increased precision of these systems often occurs at the expense of a decrease in their explainability. Although we will clarify later that the relationship between both properties does not necessarily always involve an inverse correlation, the ethical debate has mainly focused on the view that the more accurate AI is, the less we are able to understand the mechanism by which the algorithmic outcome (and the clinical conclusion) is reached. This trade-off has sparked an interesting ethical debate on medical AI. Alex John London has claimed that accuracy should be a more important value than explainability in the applications of AI to medicine (London 2019). He argues that the absence of causal knowledge is already a common phenomenon in some implemented medical practices—such as the use of aspirin or lithium. Similarly, London points out that empirically validating the accuracy of AI in healthcare is significantly more important than knowing the underlying factors that lead a system to produce an output. Thus, explainability is an unnecessary expectation that could misdirect the development of AI in medicine.

In this article, we argue that London’s position is problematic. We focus our critique on one area where the inexplicability of AI is of particular concern: the allocation of scarce medical resources. AI has great potential to support decisions about the allocation of scarce resources (such as organs or intensive medical services). Problems of inexplicability in this area have been underexplored in the AI ethics literature. In this respect, the contribution of our article is twofold. On the one hand, we show that, while accuracy is a fundamental value from an outcome-oriented justice perspective, explainability is an indispensable requirement from a procedural justice perspective. Some core aspirations in AI ethics that align with procedural fairness—(i.e. transparency, avoidance of bias, and accountability) suggest that being able to explain the algorithms used for allocating scarce medical resources is a requirement. Consequently, unlike London’s famous assertion that accuracy is the main value that should lead AI-based decisions in medicine, we contend that explainability also fulfils important ethical functions, especially in choices that entail distributive justice claims, and should therefore be an aspiration to be pursued in future technical developments. On the other hand, beyond this theoretical reframing, we also offer some practical guidance. As highly accurate predictive algorithms whose internal processes are inexplicable may continue to emerge in the near future, we provide a set of ethical and fairness-based suggestions for evaluating the adoption of these systems in distributive healthcare decisions.

The structure of the article is as follows. We will start with a conceptual clarification of the terms ‘accuracy’ and ‘explainability’ in medical AI. We will then expand on London’s main argument in which he advocates for the primacy of accuracy over explainability. This will be followed by a clarification of the role of distributive justice in the context of medical AI, to show how accurate performance and explainable algorithms may conflict. While we recognize the relevance of accuracy from an outcome-oriented justice perspective, we argue that explainability ensures procedural fairness, which is required for accountability, avoidance of bias, and transparency. After that, we will analyse AI-based liver allocation for transplantation as an example to show the advantages of introducing explainability as a key requirement for AI-based distribution of critical medical resources. Finally, we develop five practical suggestions for those considering the adoption of unexplainable algorithms for supporting allocation decisions in healthcare.

## Clarifying the concepts and the debate

AI-based medicine sparked an increasing number of bioethical controversies and philosophical debates, including the challenges raised by accurate AI applications in healthcare that lack explainability. We shall start by clarifying the terms ‘accuracy’ and ‘explainability’.

The future of high-performance medicine will probably rely on the synergy between human and AI interaction (Topol 2019). In high-income countries, AI has the potential to become an integral part of daily medical practice in a wide range of domains—such as radiology, pathology, dermatology, critical care, ophthalmology, cardiology, gastroenterology, mental health, and so on. One of the main appeals of advanced machine learning algorithms is that they offer substantial predictive power, diagnostic accuracy, and more tailored drug prescriptions. The clinical applicability and efficacy of AI-tools certainly require further validation, but some algorithms have already proved to outperform human specialists in disease detection, speed of interpretation, risk estimation for readmission and mortality, screening of scans, and X-ray image classification. As Eric Topol explains, in the case of AI, the neural net interpretation is contrasted with physicians’ evaluation using a plot of true-positive versus false-positive rates, for which the area under the curve is used to express the level of accuracy (Topol 2019).

In this context, *accuracy* commonly refers to the achieved preciseness in the performance of medical diagnostic or prognostic tasks. We have, on the one side, precision, which is the ratio of true positives to the number of true and false positives. On the other side, *recall* (also called *sensibility*) is the relation between true positives and the number of true positives and false negatives. The greater accuracy of AI applications in healthcare is forged from the training of algorithms based on huge amounts of data. Algorithmic training in a vast trove of inputs enables the development of a remarkable capability for pattern recognition. Importantly, AI algorithms can not only provide accurate outputs but also lead to clinical recommendations that are based on masses of data that are unmanageable for humans. Overcoming the human limitation in processing a great number of variables, and the remarkable precision of clinical outputs offered by these algorithms are two of the greatest benefits of the accuracy of AI in medicine.

But, as the saying goes, all that glitters is not gold. Sometimes the price to be paid for greater accuracy is the loss of explainability. Accuracy involves a degree of complexity that turns the means by which the output was reached intractable. This is a particular challenge for various deep-learning models in medicine. While the designers may understand the architecture of the AI system that processes and classifies the data, it is often impossible to explain how and why a particular outcome is given (London 2019). Hence, the operation of the evolving and self-trained algorithms may derive into a “black box” that makes the decision process inscrutable to humans. In short, accurate AI-based clinical estimates may often lack substantial explainability. In many cases, moreover, the degree (or absence) of explainability can be proportional to the accuracy of the predictions. Consider the following insightful fragment:

There is an inherent tension between machine learning performance (predictive accuracy) and explainability. Often the best-performing methods such as deep learning are the least transparent, and the ones providing a clear explanation (e.g., decision trees) are less accurate (Holzinger et al. 2019).

There is, however, an important caveat here. Greater accuracy does not *necessarily* imply a decrease in the explainability of the system. While this is something that happens in many developments, it does not mean that it is not technically possible to obtain very precise and at the same time highly explainable systems. In fact, there are recent examples where satisfactory levels of both properties have achieved in medical applications, for example, using capsule networks (LaLonde et al. 2020; Gulum et al. 2021). The tension between accuracy and explainability may thus not be so inherent in the future. As we shall see below, the fact that both characteristics are not always mutually exclusive in the rapidly evolving technical domain is an important nuance for the ethical debate.

What does explainability mean, anyway? Explainability is an epistemic concept that refers to the possibility—and, more often, the difficulty—to know how and why an AI algorithm has yielded a particular output. It answers the question of *how does it work?* in terms of intelligibility (Floridi et al. 2018). The know-how and know-why notions relate to the development of a causal understanding of output production. In that sense, explainability can be defined as “the ability to understand and evaluate the internal mechanism of a machine, algorithm, or computational process in human terms” (Cutillo et al. 2020) or, more exactly, the property of an AI system that enables the ability of an agent to understand its mechanisms. Similarly, explaining entails providing “causes of observed phenomena in a comprehensible manner through a linguistic description of its logical and causal relationships.” (Holzinger et al. 2019) London, in a similar vein, recalls an Aristotelian definition: “For Aristotle, explanations are logical arguments in which the particular to be explained is subsumed under a more general set of claims that clarify the causal factors responsible for generating the particular.” (London 2019, p. 16) In other words, explainability would exist when the causal relationships found in the model and their reasons or dynamics are known. However, explainability, as well as accuracy, is not a fixed or binary property, but a gradual one—both being sometimes measured in percentages. In the clinical setting, broadly speaking, health professionals can explain in more detail the output if they are able to understand and logically communicate how and why the AI support system has reached a particular result.

Explanations can be subject-centric or model-centric (Watson et al. 2019a; Tsamados et al. 2020). The former refers primarily to the audience of the explanations, that is, the ultimate recipients of AI-based decisions and the targets for which explanations can be adjusted. The latter refers to explanations of the model independently of the target of explanations. These two approaches are not entirely independent of one another. For instance, the black box problem arises when it is not possible to know, not even for the developers of the algorithm, the inner logic that has led from the processing of the data to the corresponding result (Vayena et al. 2018). Certainly, it can be of great *scientific* interest to the developers to know how and why the deep learning model has reached a particular output, as explanations have epistemic value (Robbins 2019; Durán 2021).

Although explanations can be thus epistemically significant to persons, in this article we are concerned about the ethical implications of (un)explainability. The approach to explainability that prevails in the clinical setting is the subject-centric one. Explanations are due to patients. Suppose that, as some have suggested, those affected by the AI-based clinical decision support systems are entitled to request an explanation (Robbins 2019). Such explanations are not due to patients because of their epistemic value, but because of their ethical one: clinical decisions are morally relevant and require justifications—they must not be arbitrary—because they affect patients’ health and well-being.

Consequently, the relevance of explainability can be advocated (and challenged) on ethical grounds. Explainability has been considered, for instance, one of the core principles of AI Ethics. According to Floridi et al. (2022), the justification of the principle of explainability is not only defended by the significance of its epistemological facet (i.e., to interpret and understand how the system works), but also from the ethical need for holding accountable the system for the way it works. Hence, explainability is not described in terms of causal knowledge but intertwined with transparency of the internal processes of the AI models and their underlying assumptions that can reproduce societal biases and systemic inequalities. Moreover, Amann et al. stated that unexplainable AI-based clinical decision support systems threaten core ethical values in medicine (including the traditional bioethical principles of autonomy, beneficence, non-maleficence, and justice) and may even lead to deleterious consequences for individuals as well as for public health (Amann et al. 2020). For instance, if erroneous clinical decisions based on unexplainable algorithms have a detrimental impact on patients’ well-being or if they lead to widening existing socially rooted health injustices, ethical qualms will inevitably arise. In addition, as Char et al. show, the safety of any healthcare application depends upon the ability to inspect it to see the mechanisms at work and understand how the application might fail (Char et al. 2020). Non-inspectable AI systems may sometimes pose a risk of harming patients and so questions may arise about the responsibility of the algorithms in these cases, which may create, in turn, a significant backlash against AI. (Needless to say, an adverse societal reaction may also occur if the algorithmic predictions are not accurate enough). This backlash may consist, among other things, in discouraging public approval and a lack of trust in unexplainable systems that makes patients, health professionals, and society reluctant to these AI applications.

Still, some authors have compellingly argued that explainability is not always a necessary aspiration in medical AI (London 2019; Robbins 2019). They question whether the requirement of explainability is too demanding and whether it is consistent with how other medical practices are valued. In some way, the rise of the explainable artificial intelligence (XAI) debate in health practices makes us reflect on the broader epistemological foundations and ethical goals of medicine. Among these authors, the view presented by London is of particular interest (London 2019). London rightly reminds us that uncertainty and incompleteness are pervasive characteristics of medical knowledge. The absence of a thorough understanding of the underlying causal mechanisms in the pathophysiology of some diseases or specific drug recommendations pervades medical decision-making. Physicians cannot always explain how and why a particular intervention works, without this being consequential for their practice or their patients’ entitlements. Consequently, as London remarks: “decisions that are atheoretic, associationist, and opaque are commonplace in medicine.” (London 2019, p. 17) These reflections are very relevant because they make us question what we should value in the application of AI in healthcare settings. According to London, what is of utmost importance in decisions based on AI systems is accuracy—defined as the knowledge of particular facts that are proven and supported by empirical evidence. If the results are accurate,^1^ and if it is possible to empirically verify that accuracy, explainability (which would derive from general principles that explain these particularities) is then dispensable. Standard clinical models show regularities and patterns in the data introduced through associations, but do not directly track causal relationships. Therefore, requesting explainability from machine learning techniques would be too demanding and inconsistent. The requirement of explainability may in some cases be even deleterious, as the demand for more explainability from some deep-learning algorithms may reduce their accuracy (London 2019, p. 15).

Likewise, Scott Robbins argues that not all medical decisions need explanations. We routinely rely on pharmacological treatments that we do not know how they work, but we know they work (e.g., lithium, the same example given by London). Robbins acknowledges that explainability may sometimes be useful, but he denies that the principle of explainability should be required across the board (Robbins 2022, p. 497). The requirement of explainability is therefore not always *ethically* necessary.

After these preliminary clarifications, it should be clearer that the debate we are engaging with relates to the ethical tensions involved in the trade-off between accuracy and explainability in AI-based medicine. In the next section, we will focus on the prospective impact of AI in the allocation of scarce medical services and resources. We will suggest that some concerns related to distributive justice, which have remained mainly underexplored in XAI debates, can make us rethink the ethical requirement of explainability in AI medicine.

## Distributive justice and medical AI

Distributive justice theories analyse how to fairly allocate socially valuable goods and resources. Resource constraints in medicine limit the capacity to meet existing demands and needs, resulting in a wide variety of ethical, political, and economic controversies about how to allocate scarce health-related resources. In this section, we shall address how the tug-of-war between accuracy and explainability involves a discussion of distributive justice. We will engage in this debate from the perspectives of outcome-oriented justice and procedural justice. We argue that whereas accuracy is mainly relevant from outcome-oriented stances, explainability is a requirement for procedural fairness accounts. We finally claim that distributive decisions supported by AI should be based on ‘comprehensive outcomes’, to try to reconcile the ethical values of explainability and accuracy in the upcoming development of AI.

*Outcome-oriented justice* highlights that achieving fair effective results is the main criterion by which distribution justice can be assessed. Looking for effective outcomes is measured by the ability to bring about a beneficial state of affairs: justice is promoted when valuable results are fairly materialized in the real world. Various theories adopt an outcome-oriented account when discussing distributive justice issues. Consequentialism and its utilitarian variants are probably the most prominent examples. Utilitarianism aims to maximize the best consequences, yielding the greatest amount of good for the greatest number of people (Bentham 1789; Mill 1863). In medical debates, utilitarian positions commonly underscore the need for making good and fair use of scarce resources through maximizing benefits and attending to cost-effectiveness. Benefits can be measured, for instance, in terms of the maximum number of lives saved, of the number of years of life produced, and of quality-adjusted-life-years (so-called QALYs) (Williams 1985). However, if we consider non-utilitarian outcome-oriented theories of justice such as Martha Nussbaum’s version of the capability approach, benefits can then be measured in terms of the fair distribution of central human capabilities worldwide (Nussbaum 2007).

From typical outcome-oriented justice perspectives, accuracy is generally more important than explainability when AI is used to support distributive decisions. When it comes to optimizing outcomes, accuracy is of primary importance. Starke et al. have claimed—on pragmatist grounds—that “the outcome-based clinical utility of any medical machine learning program should be put to the forefront.” (Starke et al. 2021) From an outcome-oriented justice, the loss of explainability can be a reasonable price to pay when benefits outweigh the costs.

In this regard, London (2019) points out that medical practice frequently considers the experience of benefit without enough knowledge of the underlying causal system to describe how the benefits are brought about. In this sense, the opacity and lack of causal knowledge of some machine learning approaches are not so different from daily aspects of medical decision-making. The causal understanding is often incomplete, and in these cases, the empirical validation of an intervention’s practical benefits is the most important task. If the demand for explanations of how some interventions work exceeds careful empirical validation, patients, resources and progress is negatively affected, London states.

That being said, the perspective of outcome-oriented justice is not necessarily opposed to explainability, as explainability and utility are not incompatible. Being capable of explaining the AI mechanism that leads to the outcome may be useful to acquire epistemic knowledge and to check if inappropriate considerations have been taken into account in the process (Robbins 2022). Such epistemic knowledge can subsequently improve clinical decisions, making them more accurate and thereby increasing effectiveness. Regarding inappropriate considerations, explainability can be necessary to verify whether the process has considered factors that are clinically and ethically irrelevant. An essential point is, therefore, to verify whether the results are unethically biased, that is, if they unfairly favour or disadvantage particular collectives based on morally irrelevant characteristics.

At this point, outcome-oriented justice encounters at least two challenges. First, by maximizing clinical utility through the most accurate and individualized prospective outcome analysis, this can *systematically* favour or disadvantage specific groups (Starke et al. 2021). This is a recurrent critique of utilitarianism in debates on rationing, namely when the scarcity forces us to choose whom the resource will be allocated to among the patients who can benefit from it. Some groups benefit less from scarce resources due to age or worse health status, implying that the elderly or the poor (who have greater comorbidities due to social determinants of health) typically fare worse in rationing decisions (Marmot 2017; Rueda 2021). While addressing in more detail this controversy is beyond the scope of this article, it is worth mentioning that looking for maximized accuracy in the benefits is not morally neutral. Rather, that conception implicitly displays what is most desirable in the use of AI to base distributive decisions.

Second, if explainability is required to avoid unethical biases, it is because the processes (and not only the results) need to be addressed. Procedural justice accounts become important as well. In what follows, we pay attention to that dimension of distributive justice as applied to the discussions on medical AI.

*Procedural justice* emphasizes that the process on which the decisions are based is a fundamental aspect of the judgments about justice. In other words, something is fair depending on the decision-making procedure that has led to its outcome. There are different possible applications of procedural fairness approaches depending on the domain. A famous example of this conception of justice is John Rawls’ contractarian approach to political philosophy (Rawls 1971). In the legal context, conversely, procedural justice is sometimes related to having due process. Moreover, there has been an increase of the philosophical literature on procedural fairness in the development of ML, and it is generally concerned with how to operationalize fairness statistically in algorithms, how to problematize the background of underlying structural injustice, or how to suggest what kinds of processes would lead to fairer final decisions (Hedden 2021; Zimmermann and Lee-Stronach 2022; Grote and Keeling 2022). Furthermore, in public health ethics and health politics, a prominent version of procedural justice is the “accountability for reasonableness” defended by Normal Daniels. This proposal was developed to address healthcare priority setting in contexts of resource constraints (Daniels and Sabin 1998). Accountability for reasonableness—which remarks that fair processes need transparency, publicity on rationales, and open mechanisms to revise the decisions—can be applied to XAI and distributive justice in medicine. We shall focus next on the connection of explainability with transparency, avoidance of bias, and accountability.

Transparency in this context has been defined as the procedure that makes the inner workings of AI algorithms observable, informing about the underlying data, variables, and relations within the system that lead to the outcome (Durán and Jongsma 2021). Transparency is commonly opposed to *opacity*, which characterizes hidden processes that are inscrutable for humans (e.g., when it is impossible to survey the algorithm training data). Sometimes, the term ‘transparency’ is paired with explainability, as when used to refer to “the transparency of the reasons for the AI-generated decision” (Robbins 2022, p. 500). Both terms have different conceptual nuances, though. Transparency is best understood as a sort of possibility condition of interpretability and explainability. In that sense, transparency of the internal mechanisms of the model is what enables explainability in ethical terms (Tsamados et al. 2020). Importantly, transparency of the AI processes is not always intrinsically valuable and, in some cases, it can be problematic—e.g., when transparency unjustifiably endangers the privacy of patients (Watson et al. 2019b). However, transparency can be instrumentally valuable beyond enabling explainability. Transparency facilitates uncovering sources of biases (Starke et al. 2021), accountability (Coeckelbergh 2020), which is highly related to safety in the clinical setting (Char et al. 2020; Felder 2021; Tsamados et al. 2020, Yoon et al. 2021), and the trustworthiness of the system (Vayena et al. 2018; Char et al. 2020; Tsamados et al. 2020; Durán and Jongsma 2021).

Explainability and transparency are important to avoid biases in AI. Biases are one-sided tendencies or systematic deviations or errors (Moseley 2019; Amann et al. 2020; Char et al. 2020; Starke et al. 2021; Tsamados et al. 2020). The term ‘bias’ is commonly employed pejoratively as it usually hinders our epistemological and ethical goals. At the epistemological level, biases lead us away from truth; in the normative domain, they lead us away from justice. Biased data can be harmful, as they lead to unjustified bad clinical consequences for some patients. There are various types and sources of biases (and possible solutions for them) in medical AI (Vayena et al. 2018; Chorás et al. 2020; Cutillo et al. 2020). For instance, AI can lead to biased outcomes when the model has operated with unrepresentative samples, inaccurate or limited training data sets, skewed inputs given by human operators in the labelling of samples, proxies that hide variables connected to social identities such as race, gender or social class. The problem of bias, nevertheless, is not solved by simply trying to assess algorithmic performance across diverse demographics. Technology-centred solutions are limited when they neglect that biases are also a sociopolitical issue related to underlying health inequities in society (Pot et al. 2021). Biases can surreptitiously lead to favouring or disadvantaging particular social groups in contexts of historical discrimination (Char et al. 2020; Moss and Metcalf 2020), which can lead AI to reproduce societal prejudices and systemic inequalities, or even reinforce discriminatory practices. An opaque or unexplainable procedure prevents the verification of whether the decision is free from inappropriate considerations and unethical biases (Robbins 2019, pp. 497–8). Physicians and patients are thus not only concerned about the mere outputs but also about the characteristics and features on which such results are based (Amann et al. 2020). Explainability plays a crucial role in facilitating output accountability, that is, following Floridi et al., the sense in which someone is responsible for the way the AI system works (Floridi et al. 2018).

Explainability can also be fundamental for the ascription of moral responsibility, which is also related to accountability. The ascription of moral responsibility is a daunting challenge in scenarios with multiple factors and actors that influence the decisions. Suppose that AI-based clinical decision support systems gain a key role in tragic distributive dilemmas—e.g., when rationing implies denying to a patient a life-saving medical resource. We think that inexplicability—and the resulting lack of accountability—would be especially problematic in those cases because the denial of the resource may result in the death of the non-selected person. Of course, patients could also complain about the use of more explainable but less accurate algorithms, as far as low precision may lead to misallocating resources. But the point here is that the lack of transparency and explainability may undermine scrutability**,** making it difficult to trace the moral responsibility for the decisions that have been made (Tsamados et al. 2022). Insofar as explainability involves understanding the interconnected causal steps behind an output, it is related to the re-traceability of the chains of events that is fundamental to accountability judgments. Therefore, having access to the steps an algorithm followed and being able to explain them is useful to assign responsibilities in the event of bad consequences happening. This is of particular interest to the stakeholders. From an ethical checking perspective, it is a good governance mechanism to inform affected individuals how an algorithm-based decision was reached (Mökander and Floridi 2021). Moreover, the quest for accountability is not just a matter of settling responsibilities when bad outcomes occur. As long as algorithms begin to silently structure the allocation of medical goods, the firms that develop them must be accountable because otherwise they will be incentivized to create progressively more complex and inscrutable programs (Martin 2019).

Finally, we shall warn that the two approaches to justice that have discussed display legitimate interests that are not always opposed to each other. Presenting the choice between maximization of benefits *or* fair procedures as a false dichotomy would be unsatisfactory, since both are relevant aspirations of justice for many, including us. We also believe that there are theoretical examples that can help us to build bridges between outcome-oriented and procedural perspectives.

Consider Amartya Sen’s distinction between “culmination outcomes” and “comprehensive outcomes” (Sen 1997, 2009). The former are mere outcomes that do not attend to the processes that generate them, while the latter do. Although this distinction has gone unnoticed in the realm of AI ethics, this categorization has immediate consequences. Giving value to comprehensive outcomes requires us to seek not only precise results but also to pay attention to the processes that produce them, where we believe that explainability should occupy an important role. Thus, we think that, ideally, a relevant normative aspiration for the fair distribution of scarce resources through AI is the pursuit of comprehensive outcomes. In other words, the ethical ideal guiding developers of distributive AI algorithms in healthcare should seek to maximize both the accuracy of the predictions and the explainability of their processes.

The problem lies, as we have shown in the previous section, in that in the practical current and near-state developments of AI it is often difficult to reconcile accuracy with explainability. So, this ideal of seeking comprehensive outcomes may seem difficult to realize in the short term, although it is not technically impossible. Precisely, the fact that there are already some successful examples in terms of accuracy and explainability (as we mentioned in the previous section), reinforces our thesis that future efforts in AI design should try to combine both aspirations for the allocation of scarce health-related resources. This, of course, does not exempt us from continuing to consider real-world examples where the ethical trade-off is present. Therefore, in the next section, we will approach a practical case in which the analysis of the trade-off between accuracy and explainability can illuminate this kind of distributive challenge on medical AI.

## The case of AI-based liver allocation

AI can influence the allocation of scarce healthcare resources in a variety of domains, such as ICU prognosis or organ distribution. In this section, we briefly address the case of AI-based liver allocation among candidates for a transplant. Our purpose is to turn our previous theoretical framework into a concrete case and show the practical relevance of the trade-off between accuracy and explainability.

For the last two decades, the distribution of transplantable livers has been governed in most countries by a criterion of need/urgency based on short-term mortality prediction. In other words, priority is given to those who are at the greatest risk of dying while awaiting transplantation. This prediction is commonly made by a scoring system, the Model for End-stage of Liver Disease (MELD), which uses linear regression on three recipient variables: creatine, bilirubin, and International Normalized Ratio (Freeman 2007). The more recent introduction of suboptimal organs (from donors with greater deterioration) underscores the need of introducing donor variables into allocation decisions. The goal is to specify the risk of transplant failure and, by doing so, to maximize organ longevity. If suboptimal organs were transplanted to patients with a good prognosis, re-transplantation would probably be necessary. Conversely, if optimal organs were transplanted in patients with a poor prognosis, the patient would likely die with a functional organ that could have provided more life to another recipient. These inefficiencies in the context of chronic and increasing organ shortages are the entry point for AI tools, which are aimed at achieving more accurate post-transplant predictions (Brown et al. 2012; Ershoff et al. 2020, Wingfield et al. 2020).

The first evolution towards a more complex predictive algorithm that takes into account both donor and recipient characteristics is the Transplant Benefit System (TBS), implemented in the United Kingdom. Although not properly generated with AI models, this system uses 28 variables—7 from the donor and 21 from the recipient—to establish a better prediction of donor-recipient matching outcomes. This system aims at directing transplantable livers to the recipient where the organ can last the longest (Wingfield et al. 2020). The greater complexity of the TBS model already poses an explainability problem with respect to the previous MELD model, as the increase of variables blurs their connection with the result. However, they all have a linear relationship with the result, which makes it possible to assess the relative weight they bring to the allocation decision.

Neural networks have also been used to generate models for predicting 90-day mortality after hepatic transplantation that are expected to be more accurate than linear models (Briceño et al. 2014). These models detect nonlinear relationships that optimize organ-recipient matching and allow the liver to be directed to the pair with the best prognosis for organ survival within the recipient (Brown et al. 2012; Dorado-Moreno et al. 2017; Briceño 2020; Ershoff et al. 2020). Apart from its direct application for allocation purposes, these models have a wide range of applications as a tool to improve physician confidence in marginal organs’ usefulness and to personalize informed consent for transplantation (Wingfield et al. 2020).

In comparison with linear models, AI-based liver distribution algorithms have increased accuracy (Dorado-Moreno et al. 2017; Briceño 2020). However, these models do not allow assessing or reporting the relative weight of each of the input variables in the final prediction (Briceño et al. 2014; Wingfield et al. 2020; Briceño 2020). Although variables that should be considered irrelevant in liver distribution—e.g., patient’s place of residence, occupation, gender or ethnicity—can be excluded from the AI programming, its content may be indirectly captured by the combination of other clinical variables, thus having a certain, although not measurable, impact on the allocation decision. This involves a risk of unfairness resulting from a deficit of explainability: data that is a priori innocuous and has no understandable or justifiable relationship with transplant survival may become inadvertently decisive in the allocation judgment. And still, disregarding these variables would decrease the accuracy of the matching and may result in allocation errors.

This is an example of the tension between accuracy and explainability in the distribution of a scarce medical resource. Higher accuracy in graft survival prediction can only be obtained at the expense of losing explainability to patients and clinicians (Wingfield et al. 2020). On the one hand, AI-based liver allocation has the potential of giving accurate predictions of graft survival and helps to optimize the benefits that each organ can provide. Tailoring the allocation based on donor-recipient matching is valuable from an outcome-oriented perspective, as it helps to comply with the aspiration of efficiency.

On the other hand, the loss of explainability is worrisome from a procedural fairness perspective. Renouncing the explainability of these processes entails giving up the pretension of knowing the fundamental factors that account for and justify the output. Inexplicability makes it difficult to know whether and to what extent the model has considered variables that are deemed ethically irrelevant. The reduction of explainability menaces the aspiration of holding the model accountable in case of unethical biases or bad consequences for the recipients. Patients who are put behind on the waiting list or ruled out from organ allocation may find themselves entitled to request on what basis the allocation is grounded—especially when the decision may result in the death of the rejected recipient. This may reduce the trust in the AI-based distributive decision, which could in turn threaten public support for the whole transplantation system.

To summarize, the use of emerging AI models for hepatic transplantation offers the promise of remarkable predictive accuracy, but the inherent byproduct of this gain is an ethically expensive—in terms of procedural fairness—loss of explainability.

## Recommendations for the distributive use of unexplainable algorithms

Ideally, future developments of AI to support allocation decisions should reconcile the internal explainability of the system with a highly accurate predictive capability as far as technically possible. However, in the short term, we will probably continue to have inexplicable algorithms that may help predict benefits in distributional dilemmas. It follows from our previous arguments that using opaque algorithms to allocate scarce health resources is problematic, but not completely dismissable. The use of highly accurate but inexplicable AI systems may be ethically justifiable in some circumstances. What considerations should we make to render the use of unexplainable algorithms ethically acceptable? In this final section, we offer some recommendations to assess the ethical (in)adequacy of using unexplainable AI in the future allocation of limited medical resources. In what follows, we shall provide five key considerations:

1. *Ensuring trust through monitoring public preferences*. To avoid social disapproval and backlash against AI adoption, it is important to draw on available empirical studies on when the loss of explainability is widely disfavoured.

As in any public ethical debate, generating solid evidence on population attitudes is critical. To our knowledge, there is a significant lacuna in empirical data on people’s preferences on the issue we have been discussing, with a few notable exceptions. In a recent article, Nussberger and colleagues have shown how people have robust and positive attitudes toward both interpretability and accuracy in the application of AI. However, interestingly, when there is a direct trade-off between these two properties in high-stake contexts as in the allocation of scarce medical treatments, people tend to sacrifice interpretability in favour of accuracy (Nussberger et al. 2022). Similarly, in another recent article that used citizens’ juries, most jurors preferred to preserve a higher level of precision at the cost of reducing explainability in the case of kidney transplantation, but they value explainability more than accuracy in other contexts of AI application such as criminal justice (Veer et al. 2021).

The previous line of research is relevant for at least two reasons. On the one hand, it demonstrates that both accuracy and explainability are important for building public confidence in medical AI. But, on the other hand, it shows that the value of explainability should not be overestimated when there is a trade-off with predictive accuracy. Needless to say, ethical disputes cannot be solved in referendum mode (Savulescu et al. 2021). Yet, having empirical evidence on the majority public preferences is important in terms of assessing which technological innovations may or may not have societal backlash. In this respect, we call for more empirical research on the public attitudes about using unexplainable but accurate algorithms to distribute health resources in real-world cases.

2. *Attending context-sensitivity and resource dependency*. In forthcoming AI-based distributive judgments, the trade-off between accuracy and explainability should be context-dependent and sensitive to the resources available.

This recommendation is best understood with a couple of examples, in which we distinguish prioritisation from rationing cases. Imagine using AI to prioritise (i.e. establish the order of access to) ICU beds. Using opaque algorithms to suggest which patients should be allocated beds first (e.g., based on the prediction of worsening health status) is not so problematic if all candidates eventually access the resource. Similarly, and returning to the case of the previous section, if we have three unassigned transplantable livers and we have three or fewer compatible candidates on the waiting list, accuracy may be preferable to explainability if we seek to optimize the benefits of these organs, and because no one will be excluded. This will be different, however, in a rationing situation, where some potential beneficiaries would be denied the resource. In those situations, we should weigh more carefully the implications of using unexplainable AI. After all, context-dependency in a non-ideal world means that allocation decisions should be case-by-case, depending on the resources available and the number of potential candidates.

3. *Acknowledging that an algorithmic prediction does not equate to a distributive decision*. The use of predictive self-learning algorithms does not entail automated decisions; namely, AI intends to support human and institutional agency but should not replace it, especially when the prediction is based on inscrutable algorithmic processes.

The distinction between prediction and decision is relevant from a fairness perspective (Hedden 2021). The unfairness of the algorithmic process (relative to statistical criteria, its opaqueness, or the presence of biases) is not identical to the unfairness of the final decisions (Grote and Keeling 2022). Among other reasons, because the final decision does not have to follow the algorithmic recommendation. In addition, decoupling prediction from decision allows us to broaden the fairness-related idea of ‘process’. Then, by ‘process’ we do not simply mean the inner working of the algorithm, but the entire distributive process that goes beyond algorithmic functioning. Thus, although AI prediction may be gestated in inexplicable algorithmic processes, explainability may play a role at other points in the procedure of deciding and justifying the concrete resource allocation. When explanations of the predictive process cannot be given, explanations of distributional decisions must be provided to avoid a sense of arbitrariness.

4. *Fulfilling the institutional duty to provide explanations*. Patients affected by the use of unexplainable algorithms in distributional contexts should be able to receive, if they wish, explanations—not necessarily about the concrete algorithmic inner-working—but of the healthcare institution’s general rationale for adopting AI for supporting these assignments, of the ethical considerations in the design and training of the algorithms, and of the main ethical criteria that have been used to guide the distribution.

The fact that explanations cannot be given for the internal mechanisms of predictive algorithms does not preclude other types of explanations. Indeed, some have argued that technologically-focused approaches to explainability are limited. It is not simply a matter of providing technical explanations of the medical algorithm, but of providing institutional explanations of the reasons for its adoption (Theunissen and Browning 2022). Healthcare institutions should convey meaningful information to end-users and to medical professionals about the rationale of using highly-accurate algorithms despite their inexplicability. These explanations may be aimed, among others, at presenting the main reason for this technological adoption, justifying when waiving higher levels of explainability is acceptable in the interest of maintaining high predictive accuracy, ensuring that the company developing the algorithm took precautions to avoid unfair biases or demographic incompatibilities, and reporting on the ethical principles that have guided the assignment. Thus, health systems adopting AI can partially satisfy the value of explainability in the allocation of resources influenced by AI.

5. *Considering alternatives and measuring costs*. In assessing the use of unexplainable algorithms, the opportunity cost of not using them, the economic and environmental cost of adopting them, and the available alternatives must be taken into account.

When considering the introduction of AI for the allocation of scarce health resources, the following three issues need to be addressed. First, weighing the need and justification for AI; it must be assessed whether and how AI is a significant improvement over previous predictive systems, even if it relies on inexplicable algorithmic processes. Second, opportunity costs (that is, the value or disvalue of forgoing the use of AI to support distributional decisions) must be considered. Third, the economic and environmental costs of such systems, if they are to be adopted, must be measured and taken into account.

## Concluding remarks

We have shown that AI may increasingly generate difficult ethical challenges in the distribution of scarce medical resources, such as organ allocation, and that not all theories of justice would place the same value on accuracy and explainability. While accuracy is a fundamental value of outcome-oriented justice, explainability is an indispensable requirement of procedural fairness. As far as technically possible, we have argued that AI developments should ideally pursue ‘comprehensive outcomes’, accommodating the importance of outcomes and the processes that produce them. We hope that this article’s contribution helps to usefully reframe the debate on distributive justice in medical AI.

Furthermore, in cases where the use of highly accurate but inexplicable algorithms may be beneficial in supporting distribution decisions, we have offered five recommendations for ethically assessing the adoption of AI. These suggestions are not exhaustive, and future contributions will need to expand and refine them, but they may offer practical guidance for those considering the introduction of these ML systems in healthcare settings.

Finally, the challenges presented here require further societal deliberation. We believe that one of the key axes of the future debate should revolve around the factors of public acceptance of these AI applications. According to our argument, maximizing comprehensive outcomes could mean greater transparency, accountability, and avoidance of bias. Linking explainability to accuracy in the outcomes can contribute to generating a “virtuous circle” in AI-based medical resource allocations. Both properties are valuable since public confidence in AI is therefore at stake. And also not holding back its beneficial developments that can assist us, if aligned with prominent ethical ideals, in a fairer allocation of our healthcare resources.

## Data Availability

Not applicable.

## Footnotes

1 It may also be possible to interpret that accuracy works in London’s paper as a stand-in for beneficence, the core bioethics principle. We will return to this idea in the next section.
